# MiR-185/AKT and miR-29a/Collagen 1a pathways are activated in IPF BAL cells

**DOI:** 10.18632/oncotarget.12740

**Published:** 2016-10-18

**Authors:** Eliza Tsitoura, Athol U. Wells, Kostantinos Karagiannis, Ismini Lasithiotaki, Eirini Vasarmidi, Eleni Bibaki, Chara Koutoulaki, Hiroe Sato, Demetrios A. Spandidos, Nikolaos M. Siafakas, George Sourvinos, Katerina M. Antoniou

**Affiliations:** ^1^ Laboratory of Molecular and Cellular Pneumonology, Medical School, University of Crete, Heraklion, Crete, Greece; ^2^ Department of Clinical Laboratory Medicine, Laboratory of Clinical Virology, Medical School, University of Crete, Heraklion, Crete, Greece; ^3^ Interstitial Lung Disease Unit, Royal Brompton Hospital, Imperial College, SW3 6NP, London, UK; ^4^ Department of Thoracic Medicine, Interstitial Lung Disease Unit, University Hospital of Heraklion, Heraklion, Crete, Greece

**Keywords:** IPF, microRNA, bronchoalveolar lavage fluid, collagen, AKT, Immunology and Microbiology Section, Immune response, Immunity

## Abstract

MicroRNA signatures of BAL cells and alveolar macrophages are currently lacking in IPF. Here we sought to investigate the expression of fibrosis-related microRNAs in the cellular component of the BAL in IPF. We thus focused on microRNAs previously associated with fibrosis (miR-29a, miR-29b, miR-29c, let-7d, and miR-21) and rapid IPF progression (miR-185, miR-210, miR-302c-3p miR-376c and miR-423-5p). Among the tested microRNAs miR-29a and miR-185 were found significantly downregulated in IPF while miR-302c-3p and miR-376c were not expressed by BAL cells. Importantly, the downregulation of miR-29a inversely correlated with the significantly increased levels of COL1A1 mRNA in IPF BAL cells. Collagen 1 a was found mainly overexpressed in alveolar macrophages and not other cell types of the BAL by immunofluorescence. In view of the downregulation of miR-185, we tested the response of THP-1 macrophages to profibrotic cytokine TGFb and observed the downregulation of miR-185. Conversely, proinflammatory stimulation lead to miR-185 upregulation. Upon examination of the mRNA levels of known miR-185 targets AKT1, DNMT1 and HMGA2, no significant correlations were observed in the BAL cells. However, increased levels of total AKT and AKT^ser473^ phosphorylation were observed in the IPF BAL cells. Furthermore, miR-185 inhibition in THP-1 macrophages resulted in significant increase of AKT^ser473^ phosphorylation. Our study highlights the importance of BAL microRNA signatures in IPF and identifies significant differences in miR-185/AKT and miR-29a/collagen axes in the BAL cells of IPF patients.

## INTRODUCTION

Idiopathic pulmonary fibrosis (IPF) is a severe, under diagnosed lung disease characterized by an irreversible and progressive loss of lung capacity, rapidly leading to respiratory failure and increased mortality [1]. Survival rates from the time of diagnosis are comparable to those of inoperable pulmonary malignancies and until recently, evidence of a treatment option that could modify the disease course was lacking [1]. Recent advances in the understanding of IPF pathogenesis suggest that aberrant wound healing, defective re-epithelisation, fibroblast to myofibroblast transition, coupled to hyperplastic pneumocytes at the fibroblastic foci and excess accumulation of extracellular matrix components recapitulate the processes leading to the destruction of the lung architecture [2].

The identification of biomarkers in serum and bronchoalveolar lavage BAL is a growing field of research in pulmonary diseases [3]. BAL is a mildly invasive method, which allows the recovery of soluble and cellular components lining the alveolar epithelium. Alveolar macrophages constitute more than 70% of the cellular component in IPF which makes BAL ideal for their study [4]. In patients with idiopathic pulmonary fibrosis (IPF), the alveolar epithelium lining fluid contains mediators that alter fibroblast activity, such as CCL18, LPA, TGF-b and PDGF [5, 6]. Alveolar macrophages are regulated by these factors and participate in the perpetuation of the fibrotic process [7]. MicroRNAs, small non-coding RNAs contributing to the epigenetic regulation of gene expression, provide expression signatures characterizing epithelial and fibroblast function in whole tissue or cell type-specific studies [8]. However, the serum microRNA signature in IPF is characterized in only one study [9], with no reported BAL data.

MicroRNAs directly involved in fibrosis, myofibroblast proliferation and excessive extracellular matrix deposition include miR-21 and members of the miR-29 and let-7 families [8]. MiR-21 expression is enhanced in IPF fibroblasts and epithelial cells, as also seen in malignant disease [10]. The miR-29 family is among the first microRNAs found downregulated in fibrotic diseases of the lungs, heart and liver. MiR-29 acts through the inhibition of ECM, including collagens, as well as enzymes that regulate collagen synthesis, crosslinking and degradation [11]. Based on previous reports, microRNAs of particular interest in IPF pathogenesis miR-210, miR-185, miR-302c-3p, miR-376c and miR423-5p are differentially expressed in slow and rapid IPF progression [12].

MicroRNA expression in IPF has been extensively studied in the context of epithelial and fibroblast function using whole tissue or cell type-specific studies. However, little is known about their expression in alveolar macrophages. In the present study, we focused on the expression of the microRNAs listed above in BAL cells from patients with IPF. We observed that the miR-29/collagen1a axis was affected. Furthermore, we identified a significant downregulation of miR-185, shown to be biologically significant by an associated enhanced activation and expression of AKT.

## RESULTS

### Patient demographics

The main characteristics of the patients and lung function results for the IPF and control groups are shown in Table 1. There was no difference in gender, whereas the IPF patients were significantly older than the healthy subjects (mean age 65.4 vs. 48.2 years, respectively, p<10^−3^). Additionally, there were more current smokers in the control group, while there were more former smokers in the IPF group (p<10^−3^). Predictably, lung function tests showed impaired lung function in the patients with IPF.

**Table 1:** Patient characteristics

Demographics	Controls (N=17)	IPF (N=45)	p value
Age (years)	48.2 ± 3.2	65.4 ± 1.8	**<10^−3^**
Gender male female	13 4	37 8	0.721
Smoking status non smoker ex smoker smoker	2 3 9	16 24 2	**<10^−3^**
PY	36.0 ± 5.7	39.1 ± 7.1	0.764
**Lung Function**			
FEV1%	92.7 ± 6.0	73.7 ± 3.1	**0.009**
FVC%	94.0 ± 5.4	71.6 ± 3.3	**0.003**
FEV1%/FVC%	79.7 ± 2.0	79.5 ± 1.2	0.938
TLC%	101.6 ± 7.7	67.6 ± 2.5	**<10^−3^**
DLco%	79.2 ± 5.5	43.4 ± 2.3	**<10^−3^**
KCO%	103.6 ± 8.1	77.5 ± 3.2	**0.014**
**BAL cell count**			
Macrophages %	69 ± 8	73 ± 3	0.83
Lymphocytes %	24.3 ± 7.6	14.1 ± 2.7	0.17
Neutrophils %	3.7 ± 1.4	6.8 ± 1	0.12
Eosinophils %	0.8 ± 0.4	3.2 ± 0.6	**0.04**

Values are the means ± SEM. PY=pack years for ex and current smokers; FEV1= Forced expiratory volume in 1 second; FVC= Forced Vital Capacity; TLC= Total Lung Capacity; DLco= Transfer factor of the Lung for Carbon Monoxide; KCO= transfer coefficient.

### MiR-29a and miR-185 expression is reduced in IPF relative to control specimens

**Figure 1:**
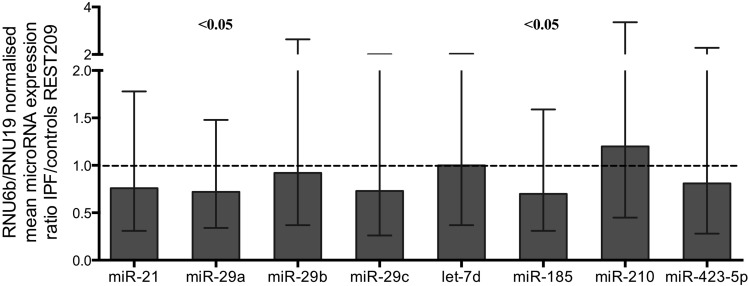
MicroRNA expression ratios in IPF relative to controls Normalized by two reference assays RNU6B and RNU19. MicroRNAs miR-29a and miR-185 showed significant difference in IPF. Boxes represent mean and error bars standard error of mean. Complete REST2009 report in supplementary file 2.

MicroRNA expression levels in BAL cells were measured by qRT-PCR and normalized using two endogenous controls, the small nuclear RNA RNU6B and small nucleolar RNA RNU19. There was a statistically significant downregulation of miR-29a and miR-185 in IPF, compared to controls following analyses using REST2009 software (Figure 1, Table 2). MiR-29a and miR-185 as well as miR-29c, miR-21, let-7d, and miR-423-5p showed significant downregulation according to Wilcoxon Signed Rank test (Table S2). MiR-302c-3p and miR-376c were not expressed by the BAL cells.

**Table 2:** microRNA expression ratios in IPF relative to controls

	Expression Ratio^a^	SE range	P^b^
miR-21	0.76	0.31-1.78	0.11
**miR-29a**	**0.72**	**0.34-1.48**	**0.045**
miR-29b	0.92	0.37-2.64	0.74
miR-29c	0.73	0.26-2.01	0.20
let-7d	1.00	0.37-2.03	0.99
**miR-185**	**0.70**	**0.31-1.59**	**0.040**
miR-210	1.20	0.45-3.36	0.40
miR-423-5p	0.81	0.28-2.28	0.33
miR-302c-3p	NE	-	-
miR-376c	NE	-	-

a Expression Ratio represents the concentration of microRNA of interest divided by the geometric mean of the concentrations of the two reference assays RNU6B and RNU19. Concentration of each microRNA equals the efficiency of the PCR reaction to the power of the average Ct of controls minus the average Ct of IPF patients.

b Probability of alternate hypothesis that difference between sample and control groups is due to chance according to REST2009. SE standard error, NE: Not expressed. Complete REST2009 report in supplementary file 2.

In IPF, fibrosis and progression-related microRNAs were largely co-expressed, as demonstrated by the strong positive correlations shown in Table 3. In particular, miR-185 expression strongly positively related with the expression of let-7d, miR-29a, miR-29b, miR-29c, and miR-21.

**Table 3:** Spearman's correlation of microRNA expression in IPF BAL

	miR- 29b	miR-29c	miR- 21	let -7d	miR-185	miR-210	miR- 423-5p
**miR-29a**	0.65 (<10^−5^)	0.64 (<10^−5^)	**0.78** **(<10 ^−9^)**	0.54 (0.0001)	0.64 (<10^−5^)	0.44 (0.002)	0.59 (<10^−4^)
**miR-29b**		0.60 (<10^−4^)	0.68 (<10^−6^)	0.42 (0.005)	0.66 (<10^−5^)	NS	0.37 (0.01)
**miR-29c**			0.54 (0.0001)	0.44 (0.0001)	0.65 (<10^−5^)	0.49 (0.0005)	0.57 (<10^−4^)
**miR-21**				0.54 (0.0001)	0.63 (<10^−5^)	0.40 (0.007)	0.54 (0.0001)
**Let-7d**					**0.71** **(<10^−7^)**	0.41 (0.005)	0.40 (0.007)
**miR-185**						0.42 (0.004)	0.56 (<10^−4^)
**miR-210**							**0.76** **(<10^−8^)**

Values are Rs correlation coefficient and in parentheses are p values. NS= not statistically significant

### Reduced microRNA expression correlates with disease severity and eosinophil infiltration

Diffusing capacity of the lungs for carbon monoxide (DLco), which is impaired in IPF, positively related to the reduced levels of miR-29a levels (Rs = 0.33, p<0.05) and miR-29c levels (Rs = 0.36, p=0.03). MiR-185 expression was not significantly linked to DLco (Table S3). Additionally none of the microRNAs tested was associated with patient survival.

The presence of rare eosinophils in the BAL is common in IPF (Table 1) and has been related to disease severity. BAL eosinophil percentages were strongly negatively related to the expression of miR-210 and were also negatively related to miR-423-5p, let7d, miR-21 and miR-29a expression but not to miR-185 (Table 4). These relationships remained significant in separate stepwise multiple linear regression model, in which each individual micro-RNA was examined in a separate model, with age, smoking status, DLco levels and BAL cellular profiles included as covariates.

**Table 4:** Correlations of microRNA expression with eosinophil % within IPF group

MicroRNA	Spearman's		Statistical significance in multiple linear regression models^a^
	r	p	p
**miR-21**	**-0.32**	**0.03**	**<0.001**
**miR-29a**	**-0.34**	**0.03**	**0.02**
miR-29b	0.12	NS	NS
miR-29c	−0.25	NS	NS
**let-7d**	**-0.42**	**0.005**	**<0.001**
**miR-210**	**-0.66**	**<0.0001**	**<0.001**
miR-185	−0.21	NS	NS
**miR-423-5p**	**-0.55**	**0.0001**	**0.03**

a The independent relationships between microRNA levels and BAL eosinophil percentages in stepwise multiple linear regression models, with age, smoking status, DLCO levels, BAL neutrophil levels and BAL lymphocyte levels included as covariates.

BAL lymphocyte levels were positively related to miR-210 (Rs = 0.40, p<0.01) and miR-423-5P (Rs = 0.44, p<0.005) but relationships were not significant in multiple linear regression models. There were no significant relationships between microRNA expression and BAL neutrophil or macrophage levels.

### BAL cells from IPF patients displayed a pro-fibrotic profile with miR-29a downregulation and collagen 1a overexpression

**Figure 2:**
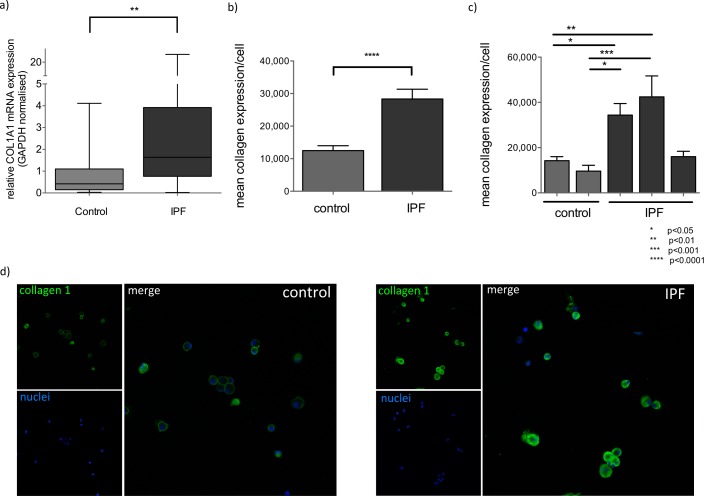
Collagen 1 expression is elevated in IPF BAL a) Expression of COL1A1 mRNA, normalized by GAPDH in control and IPF samples, (**:p<0.01, p value, Mann-Whitney test). Box plots represent median, 25 and 75 percentiles and error bars correspond to minimum and maximum values. b) Quantification of mean collagen 1a1 expression per cell in alveolar macrophages expressed by mean pixel intensity multiplied by positive pixels (excluding background)/cell analyzed collectively from 3 IPF and 2 control measurements. (****:p<0.0001 unpaired t-test). c) Mean collagen 1a1 expression per cell per BAL sample (*:p<0.05, **:p<0.01, ***:p<0.001 ordinary one way ANOVA). Error bars represent standard deviations. d) Representative confocal images of Control and IPF BAL cells stained with anti-Collagen 1a1(green) and DNA specific dye (blue) at 40x magnification.

MiR-29a downregulation has been associated with increased collagen expression in IPF tissues and animal models of fibrosis however, it is not known if this also occurs in alveolar macrophages. We examined collagen expression by RT-PCR and immunofluorescence and showed that COL1A1 mRNA levels were significantly increased in IPF BAL cells, compared to controls (p=0.003) (Figure 2a). In IPF, COL1A1 mRNA levels inversely correlated with the expression of miR-29a (Rs = −0.42, p = 0.005). Collagen1a1 protein levels were measured by immunofluorescence. Alveolar macrophages were positively stained with anti-collagen1a1 antibody both in controls and IPF BAL samples. However, in IPF BAL samples alveolar macrophages showed significantly higher cytoplasmic collagen staining than the controls (Figure 2b-2d).

### MiR-185 inhibition results in AKT activation in macrophages

**Figure 3:**
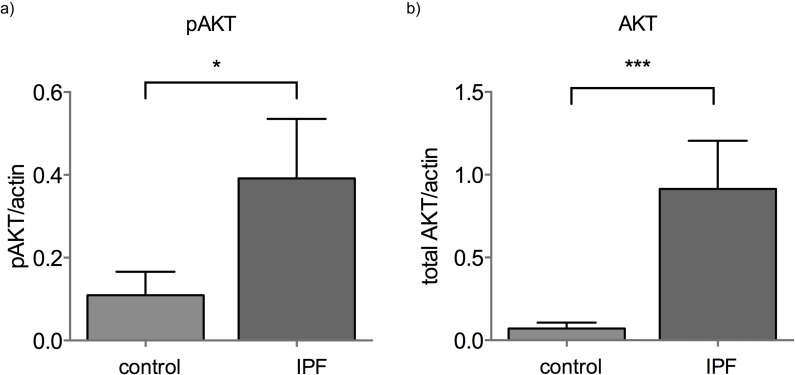
AKT and pAKT levels are elevated in IPF BAL a) AKT1 and b) pAKT^ser473^ protein levels normalized by actin in control and IPF BAL samples (*:p<0.05, ***:p<0.005 p value, Mann-Whitney test).

MiR-185 was significantly downregulated in IPF BAL cells. The miR-185 reported mRNA targets, AKT1, DNMT1 and HMGA2 were not upregulated (Figure S1a-c) nor did they show any correlation with miR-185 expression. However, in IPF, AKT protein levels are strikingly elevated and activated as shown by the pAKT^ser473^ levels (Figure 3, Figure S2). It is noteworthy that in contrast to miR-185, miR-21 and let-7d inversely correlated with their reported target HMGA2 in the BAL (Rs = −0.36, p = 0.016 for miR-21 and Rs = −0.347, p=0.023 for let-7d).

**Figure 4:**
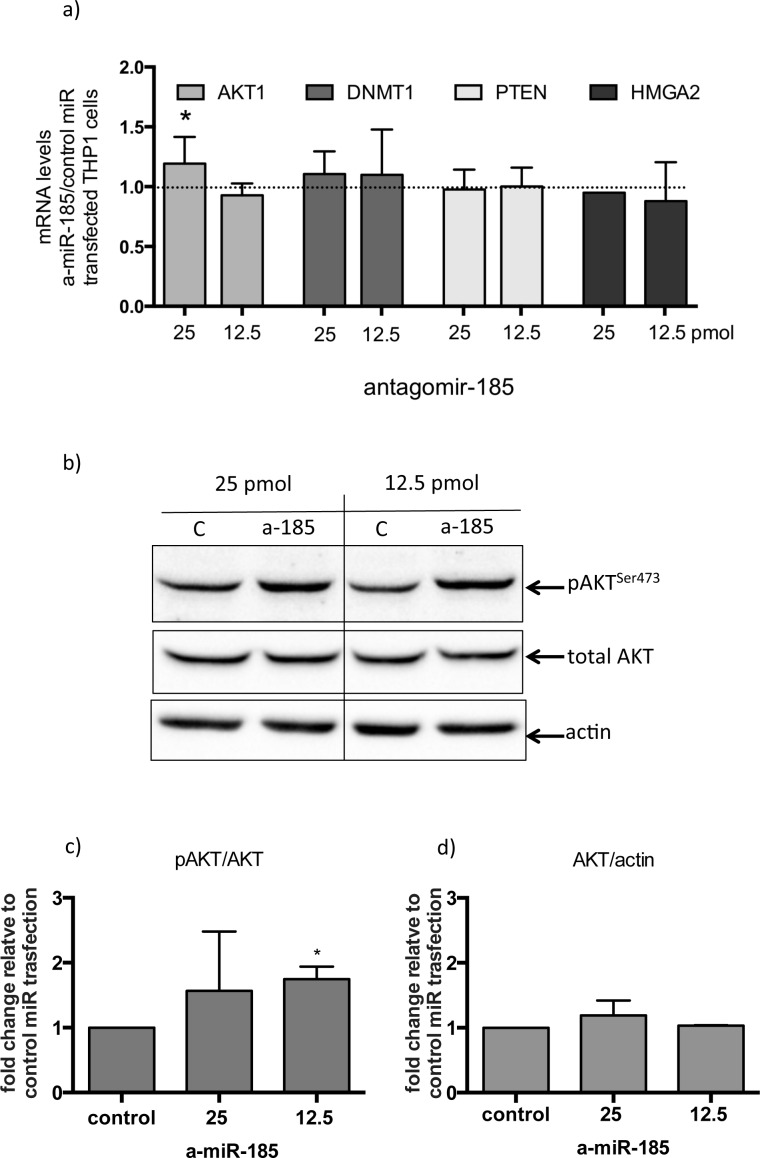
Inhibition of miR-185 in THP-1 cells treated with PMA, results in AKT activation **a**) fold changes in expression of AKT1, DNMT1, PTEN and HMGA2 mRNA in 25 or 12.5 pico-molar concentrations of antagomiR-185 RNA transfected THP1 cells relative to control transfection. (*:p<0.05 of one sample t-test). **b**) pAKT^ser473^ and total AKT1 protein levels in 25 or 12.5 pmol concentrations of control miR (c) and antagomiR-185 (a-185) transfected THP1 cells. **c**) and **d**) Densitometry analysis of three independent transfection experiments of pAKT^ser473^ relative to total AKT1 and total AKT relative to actin respectively. (*:p<0.05 of one sample t-test). Boxes represent mean and error bars are standard deviations

A specific fluorescent miR-185 antagomir was used in order to test the effects of miR-185 inhibition on macrophages. As a macrophage cell culture model, PMA activated THP1 cells were used. Two different concentrations of control-nonspecific microRNA and the specific miR-185 antagomir were used and transfection efficiency was verified using fluorescent microscopy (Figure S3). MiR-185 transient inhibition resulted in a moderate but significant increase in AKT1 mRNA levels with no significant effect on other miR-185 direct or indirect targets such as DNMT1, HMGA2 or PTEN respectively (Figure 4a). Importantly, however, miR-185 inhibition resulted in a significant increase in pAKT^ser473^ levels (Figure 4b and 4c), while total AKT levels remained unchanged (Figure 4b-4d).

### TGFb is an inhibitor of miR-185 in macrophages

**Figure 5:**
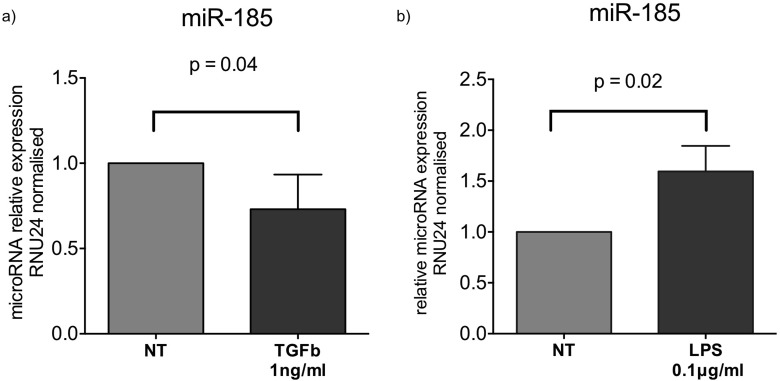
Fold changes in expression of miR-185 following **a**) TGFb1 stimulation at 1ng/ml or **b**) LPS at 0.1ng/ml of PMA treated THP1 cells for 16 hours. (*:p<0.05 of one sample t-test). Boxes represent mean and error bars are standard deviations.

We also examined the changes in miR-185 expression following exposure of PMA treated THP1 cells to fibrotic/anti-inflammatory TGFb1 or pro-inflammatory LPS stimuli. Our results showed that miR-185 was downregulated by TGFb1 at 1ng/ml following 16 hours of exposure (Figure 5a). MiR-185 was conversely upregulated by LPS at 0.1ng/ml concentration following 16 hours of exposure (Figure 5b).

## DISCUSSION

This is the first BAL study of the expression of key microRNAs involved in fibrosis, such as miR-21, miR-29a, miR-29b, miR-29c, and let-7d, as well as miR-185, miR-210, miR-302c-3p miR-376c and miR-423-5p differentially expressed in rapid IPF progression in the BAL cells of IPF patients [8, 12–14]. Two major microRNA controlled pathways were found to be differentially regulated in IPF BAL cells; the well-documented fibrogenic miR-29a/Collagen axis and the miR-185/AKT pathway, previously unrecognized in pulmonary fibrosis.

MiR-185 was significantly downregulated in IPF BAL cells relative to controls. Mir-185 was initially reported to be upregulated in rapidly progressing IPF [12] but in later studies, miR-185 downregulation was demonstrated in IPF lungs, compared to controls [15, 16]. MiR-185 downregulation is associated with the deregulation of cell cycle and cell proliferation in non-small cell lung cancer and hepatocellular carcinoma [17, 18]. Specifically, downregulation of miR-185 results in the activation of AKT via increased DNMT1 expression, leading to promoter hypermethylation and silencing of PTEN [18]. Additionally, miR-185 directly binds and degrades the 3'UTR of AKT1 mRNA [17].

In BAL cells there was no correlation between mRNA levels of AKT1, DNMT1 or PTEN and miR-185 levels. However, we observed an overexpression of AKT protein in IPF that was coupled to increased activation demonstrated by AKT^ser473^ phosphorylation. The observed increased AKT activation in BAL cells is in agreement with previously reported elevated basal levels of PI3K activity [19] and consistent also with the increased anti-apoptotic profile of IPF macrophages [20]: AKT is activated by P13K phosphorylation and subsequently functions as a serine/threonine kinase involved in multiple cellular processes including cell proliferation, inflammation, survival and glucose metabolism [21]. IPF fibroblast cultures also display a hyper activation of AKT leading to increased viability in collagen-rich matrices [22, 23]. Furthermore, a very recent study implicates AKT1 activation in the production of TGF-b by alveolar macrophages in the context of pulmonary fibrosis [24].

Importantly, AKT overexpression in IPF macrophages might also participate in the deregulation of the innate defense mechanisms of alveolar macrophages such as inflammasome activation [25] and antiviral responses [26] previously proposed by our group. AKT activation dampens the response of monocytes to LPS [27] and deletion of AKT1 in animal models of *S. aureus* lung infection results in stronger pro-inflammatory responses leading to a reduced bacterial burden [27]. Thus, increased AKT activity in IPF macrophages, observed in our study, may contribute to an increased microbial burden, which, in IPF microbiome analyses, is predictive of decline in lung function and death [28].

As the role of miR185 on AKT has not been studied in macrophages, we tested the effect of miR-185 inhibition in an *in vitro* macrophage cell culture model of PMA activated THP1 cells. The transient inhibition of miR-185 resulted in a modest but statistically significant upregulation of AKT1 mRNA and the potent activation of AKT as demonstrated by the phosphorylation of AKT^ser473^, while we did not observe an effect on DNMT1 and PTEN mRNA levels. This suggests that AKT phosphorylation by miR-185 downregulation is regulated by additional factors, which are currently under investigation. However, since miR-185 inhibition alone was sufficient to activate AKT in macrophages *in vitro*, we propose that miR-185 downregulation observed in the BAL cells may contribute to the activation of the AKT signaling pathway in IPF.

In BAL fluid cells, miR-29a was significantly downregulated. Additionally, the reduction in miR-29a and miR-29c expression was significantly associated with reduced DLco levels. Members of the miR-29 family are considered major regulators in pulmonary fibrosis and may have a future therapeutic role, based on evidence of anti-fibrotic effects with miR-29 administration in mice [37]. Furthermore, miR-29a downregulation correlated with the overexpression of the collagen gene COL1A1 suggesting that the miR-29a/COL1A1 pathway is also active in IPF BAL cells, as previously demonstrated in IPF tissues [11]. Mir-29a downregulation and COL1A1 upregulation is consistent with the skewing of macrophages towards a previously reported alternative-activation and pro-fibrotic phenotype [7, 19, 29, 30]. Regarding the expression of collagen by macrophages in particular, a previous study in mice showed that upon AEC II-induced apoptosis, exudate macrophages and their precursors, Ly-6c^high^ monocytes, accumulate in the affected areas, adopting an alternative activation with IL-13, TGFβ and, notably, Collagen 1 expression [30]. Therefore, provided that our results are validated in other IPF cohorts, BAL miR-29a/COL1A1 expression levels may represent an informative IPF biomarker.

Additionally, we observed strong positive correlations between levels of miR-29 family members and miR-185 expression. In IPF fibroblasts, the negative feedback signaling induced by polymerized collagen fibers on ECM production and cell proliferation is defective and concomitantly miR-29a is abnormally further downregulated coupled to collagen overexpression [22]. In alveolar macrophages from IPF patients, exogenous collagen treatment also results in the activation of PI3K activity suggesting a positive feedback loop in IPF by collagen through the PI3K/AKT pathway. It is conceivable that miR-185 downregulation participates in the perpetuation of AKT over-activation that may further enhance miR-29 family downregulation and collagen overexpression. A recent report has shown that miR185 directly targets Collagen V mRNA [16]. Our results show that miR-185 expression is also reduced by TGFb1 similarly to fibrosis related microRNAs miR-29a [31] and Let-7d [32]. Conversely pro-inflammatory stimulation of macrophages with LPS resulted in an upregulation of miR-185 expression. This is consistent with the upregulation of miR-185 in acute lung injury models [33]. Although it is not known whether miR-185 and miR-29 are transcriptionally co-regulated, there is evidence to suggest that histone deacetylation by HDAC4 regulates both molecules [33, 34], while HDAC4 is also downregulated by TGF-b [35]. Thus, the miR-185 and miR-29a pathways may converge in AKT activation following TGF-b signaling.

Our results demonstrated that in the BAL cells, in agreement with previous studies using tissue samples [12], there was an overall decrease in the transcription of microRNAs. Interestingly the reduction in the expression of miR-210, miR-423-5p, let7d, miR-21 and miR-29a in BALF cells correlated with the infiltration of eosinophils. In IPF, elevated bronchoalveolar lavage eosinophil counts and ECP levels are associated with more rapid progression of disease [36, 37]. The higher BAL eosinophil levels observed in fibrotic idiopathic interstitial pneumonia, when compared with scleroderma lung, a less progressive disorder, indicate that an eosinophilic influx may be linked to the pathogenesis of the disease [38].

Notably, miR-21 previously reported to increase in IPF lungs [13, 14], was actually downregulated in BAL cells. Our results therefore highlight similarities in IPF microRNA profiles between the BAL cellular component and lung tissue as demonstrated by miR-29a downregulation but also striking differences such as the reduced expression of miR-21. These results suggest that BAL microRNA signatures are unique relative to the whole tissue signatures, albeit not surprisingly as the cellular composition of the two compartments is distinct. This is probably reflected in the reduced expression of miR-21 in the BAL cells. MiR-21 upregulation in macrophages/monocytes is commonly associated with pro-inflammatory stimulation by viruses, bacteria and other “danger associated molecular patterns” and plays an important role in the orchestration and eventually containment of the innate immune process [39]. The lack of miR-21 upregulation in the BAL cells is therefore in accordance with increased pro-fibrotic as opposed to pro-inflammatory macrophages in IPF. Since miR-21 is also an indirect regulator of AKT activity through the targeting of PTEN further work is needed in order to delineate the complex cross talk between miR-21 and AKT activation in alveolar macrophages in IPF. Our results show that BAL cell transcriptional signatures merit further characterization with more high-throughput approaches, a major limitation of our study, in order to validate not only its diagnostic and prognostic value but also the putative role of alveolar macrophages in the pathogenesis of IPF.

Although current thinking in IPF pathogenesis emphasizes the role of epithelial-fibroblast cell interactions, innate immune cells, such as macrophages neutrophils and eosinophils play a paramount role in the deregulation of the wound healing process. Our study provides novel evidence of the involvement of the miR-185/AKT pathway in IPF BAL cells, and provides support for the use of miR-29a and miR-185 as BAL IPF biomarkers.

## MATERIALS AND METHODS

### Human Subjects

BAL samples from sixty-two subjects, consisting of patients with IPF (n=45) and healthy control subjects (n=17) were analysed. Twenty-seven IPF patients were diagnosed at the Interstitial Lung Disease Unit, Royal Brompton Hospital, Imperial College, London, UK and the remaining eighteen IPF patients and seventeen healthy control subjects were recruited from the Department of Thoracic Medicine, University Hospital of Heraklion, Crete, Greece. Patients and control subjects were classified as current smokers, former smokers (defined as having smoked a minimum of one cigarette a day for a minimum of 1 year, stopping at least 6 months before presentation) or non-smokers. All patients were evaluated with complete pulmonary function tests (PFTs), performed within 1 month of CT, including spirometry, measurement of lung volumes and diffusion capacity. Spirometry, lung volumes using the helium-dilution technique and DLco (corrected for haemoglobin) using the single breath technique were performed using a computerized system (Jaeger 2.12; MasterLab, Würzburg, Germany). Predicted values were obtained from the standardized lung function testing of the European Coal and Steel Community, Luxembourg (1993). Pulmonary function tests, FEV_1_, FVC, and DLco corrected for hemoglobin concentration, expressed as percentages of the predicted normal values. The diagnosis of IPF was based on open or video-assisted thoracoscopic biopsy, with all biopsies reviewed by the same two histopathologists, or using ATS/ERS clinical and HRCT criteria [40]. In accordance with the aforementioned criteria, any known cause of pulmonary fibrosis, such as a systemic connective tissue disorder, was excluded by both immunologic screening and rheumatologic clinical evaluation [40]. All IPF patients were newly diagnosed and had not received previous treatment. The control subjects were patients undergoing bronchoscopy for the investigation of haemoptysis, without any overt pulmonary comorbidities and with normal bronchoscopic findings and cytology results. The study was approved by the Ethics Committees of the University Hospital of Heraklion (IRB number: 17030) and the Royal Brompton Hospital (REC reference 13/LO/0857).

### BAL cell isolation and determination of cellular composition

BAL was obtained from all patients as previously described [25, 41]. 1-1.5 million cells were homogenised in TriReagent^TM^ (MBL) for total RNA, or RIPA buffer (Invitrogen) containing protease and phosphatase inhibitors (Pierce) for Western blot protein analysis, followed by storage at −80°C. Differential cell population count was analysed following May-Grunewald-Giemsa staining as described in [41].

### microRNA and mRNA expression levels analyes

Total RNA was isolated using the mirVana^TM^ miRNA isolation kit (Ambion) with minor modifications. 350μl of TriReagent (MBL) was used for cell lysis and storage of samples, followed by addition of 350μl of mirVana^TM^ cell lysis solution and 35μl of mirVana^TM^ microRNA additive. All further steps for the isolation of total RNA were performed as recommended by the manufacturer. Quality and quantity of isolated RNA was assessed by agarose gel electrophoresis and spectrophotometry (Nanodrop) respectively. For the analysis of microRNA expression levels, 10ng of total RNA were used in reverse transcriptase and real time qPCR reactions using the TaqMan^TM^ microRNA assays (Life Technologies) and 7500 Fast Real-Time PCR system (Applied Biosystems). For gene expression analyses, 500ng of total RNA were treated with DNAfree (Ambion) for genomic DNA contamination removal, followed by 1^st^ strand cDNA synthesis using Maxima RT ^TM^ (Fermentas) and real time qPCR analysis using Maxima SYBR Green qPCRmix (Fermentas) on Mx3005P qPCR system (Agilent Technologies). Probe and primer sequences are summarized in Supplementary Table 1. RNU6B and RNU19 levels were used as endogenous controls for the normalization of microRNA expression levels in BALF samples. GAPDH levels were used as endogenous control for the normalization of mRNA expression levels in BALF samples.

### Antagomir-185 transfection of THP-1 cells

THP-1 cells were cultured in RPMI-1640 (Biosera) supplemented with 10% fetal calf serum (FCS) (Biosera) and 1% penicillin-streptomycin in a humidified incubator at 37°C containing 5% CO_2_. 5 × 10^5^ THP-1 cells were treated with PMA at 50ng/ml final concentration in 2% FCS supplemented RPMI-1640 for 16 hours, followed by addition of Lipofectamine 2000 (complexes with 25 or 12.5 pmol of miR-185 specific antagomir (Exiqon) or scramble control (Exiqon) in serum free RPMI-1640 for 4 hours. Subsequently cells were supplemented with RPMI-1640 containing 2% FCS for 24 hours prior to cell lysis for total RNA extraction or total protein analysis.

### SDS-PAGE and Western blot analysis

Total protein lysates (20ng) of BAL samples or THP-1 transfected cells were lysed with RIPA lysis buffer supplemented with protease and phosphatase inhibitors for phosphoprotein and total protein analyses. Lysates were separated in 12% SDS-PAGE, transferred to 0.45nm nitrocellulose membrane (Biorad), followed by Western blot detection of pAKT^ser473^, total AKT and b-actin, with anti-pAKT^ser473^ antibody #9271, (Cell Signaling), anti-AKT antibody # 9272 (Cell Signaling) and anti-b-actin monoclonal antibody (Chemicon). Appropriate HRP conjugated secondary antibodies (Chemicon) were used and immunodetection was performed with enhanced chemiluminescence reagent Luminata^TM^ (Millipore). Bands were visualised with the ChemiDoc XRS+ system (Biorad) and densitometry analyses were performed using Image Lab ^TM^ software (Biorad).

### TGFb1 and LPS treatments

2 × 10^6^ THP-1 cells cultured in RPMI-1640 (Biosera) supplemented with 2% FCS (Biosera) and 1% penicillin-streptomycin in a humidified incubator at 37°C containing 5% CO_2_ were treated with PMA at 50ng/ml final concentration for 16 hours prior to the addition of TGFb1 #100-21(PeproTech) or LPS (SIGMA) for the indicated concentrations and time points. Cells were subsequently lysed in TriReagent^TM^ (MBL) for total RNA extraction or RIPA lysis buffer supplemented with protease and phosphatase inhibitors for phosphoprotein and total protein analyses.

### Collagen1a1 immunofluorescence

BAL cell cytospins obtained as previously described in [41] were fixed with 4% formaldehyde PBS and labeled with anti-collagen1a1 polyclonal antibody PA2140-1 (Boster Biological Technology) followed by anti-rabbit alexa488-conjugate (Molecular Probes). Nuclei were stained with alexa-633-ToPro (Molecular Probes). Images were acquired with Leica Confocal Microscope at 40x magnification. Quantification of collagen 1a1 expression was performed with ImageJ software. Mean pixel intensity x pixels (excluding background)/cell in 4 independent fields per sample were analysed by ordinary one way ANOVA for 3 IPF BAL samples and 2 controls. Collectively IPF and control measurements were analyzed by unpaired t-tests.

### Statistical analysis of mRNA and microRNA expression

MicroRNA and mRNA expression analysis between control and IPF groups were performed using the REST 2009 (QIAGEN [42]) software following incorporation of average (duplicates) Ct values and relative efficiency values calculated using standard curves for each microRNA or mRNA assay. Furthermore, relative expression values per sample for each microRNA or mRNA assay studied were calculated by the Pfaffl method using as calibrator sample the mean of all average Ct values [42]. Analysis of expression values with lung function tests and BAL cell population percentages was hence performed with STATA software (STATA data analysis software; Computing Resource Center) and Prism 6 software. Group comparisons were made by analysis of variance, Student *t* test, Wilcoxon rank-sum test, or chi-square testing as appropriate. *P* values lower than 0.05 were considered statistically significant.

## SUPPLEMENTARY MATERIALS FIGURES AND TABLES



## Abbreviations

IPF: Idiopathic Pulmonary Fibrosis
CPI: Composite Physiologic Index
HRCT: High Resolution Computed Tomography
FEV_1_: forced expiratory volume in one second
FVC: forced vital capacity
DLco: diffusing capacity for carbon monoxide
NS: not significant
BAL: Bronchoalveolar Lavage
miRNA: microRNA
COL1A1: collagen 1a1
LPA: Lysophosphatidic acid
TGFb: transforming growth factor beta
PDGF: *Platelet-derived growth factor*
DNMT1: DNA methyltransferase 1
HMGA2: High Mobility Group AT-Hook 2
