# Supplementary material for: MiR-185/AKT and miR-29a/Collagen 1a pathways are activated in IPF BAL cells
Source: Oncotarget. 2016 Oct 18;7(46):74569–81. doi: 10.18632/oncotarget.12740 (PMC5342687; doi:10.18632/oncotarget.12740)
Supplement: Supplementary file 1 [file oncotarget-07-74569-s001.pdf]

MiR-185/AKT and miR-29a/Collagen 1a pathways are activated in IPF BAL cells

Supplementary Material

Table S1: IDs of TaqMan microRNA assays and primer sequences used for the quantification of microRNAs and mRNAs respectively

a) microRNA Assay Name	microRNA Assay ID
RNU24	001001
RNU19	001003
RNU6B	001093
hsa-let-7d	000380
hsa-miR-185	002271
hsa-miR-21	000397
hsa-miR-210	000512
hsa-miR-29a	002112
hsa-miR-29b	000413
hsa-miR-29c	000587
hsa-miR-302c-3p	000533
hsa-miR-376c	002122
hsa-miR-423-5p	002340

b) Gene Name	Primer sequences
GAPDH	F: agccacatcgctcagaca<br>R: ccaatacgaccaaatccgtt
COL1A1	F: gggattccctggacctaaag<br>R: ggaacacctcgctctcca
AKT1	F: gcagcacgtgtacgagaaga<br>R: ggtgtcagtctccgacgtg
PTEN	F: gctacctgttaaagaatcatctgga<br>R: ctggcagaccacaaactgag
HMGA2	F: tccctctaaagcagctcaaaa<br>R: acttgttgtggccatttcct
DNMT1	F: tttctgatgaaaaagacgaggat<br>R: tttctccgttggttctttgg

Table S2: Median microRNA expression ratios in IPF relative to controls.

MicroRNA	Median Expression	Interquartile range	P[a]
**miR-21**	**0.59**	**0.49-0.80**	**<0.0001**
**miR-29a**	**0.72**	**0.46-0.90**	**<0.001**
miR-29b	0.95	0.50-1.28	NS
**miR-29c**	**0.58**	**0.36-0.85**	**<0.001**
**let-7d**	**0.78**	**0.48-1.08**	**<0.05**
**miR-185**	**0.69**	**0.40-0.82**	**<0.005**
miR-210	0.81	0.51-1.47	NS
**miR-423-5p**	**0.59**	**0.42-0.85**	**<0.01**
miR-302c-3p	Not expressed		
miR-376c	Not expressed		

[a]P value of Wilcoxon Signed Ranked test , $p<0.05$ considered significant.

Table S3: Correlation of microRNA expression with DLCO % (Spearman test)

microRNA vs DLCO%	Spearman's r	p value
miR-21	0.29	0.08
**miR-29a**	**0.32**	**0.047**
miR-29b	0.18	0.27
**miR-29c**	**0.36**	**0.03**
let-7d	0.27	0.09
miR-210	0.09	0.57
miR-185	0.16	0.34
miR-423-5p	0.16	0.35

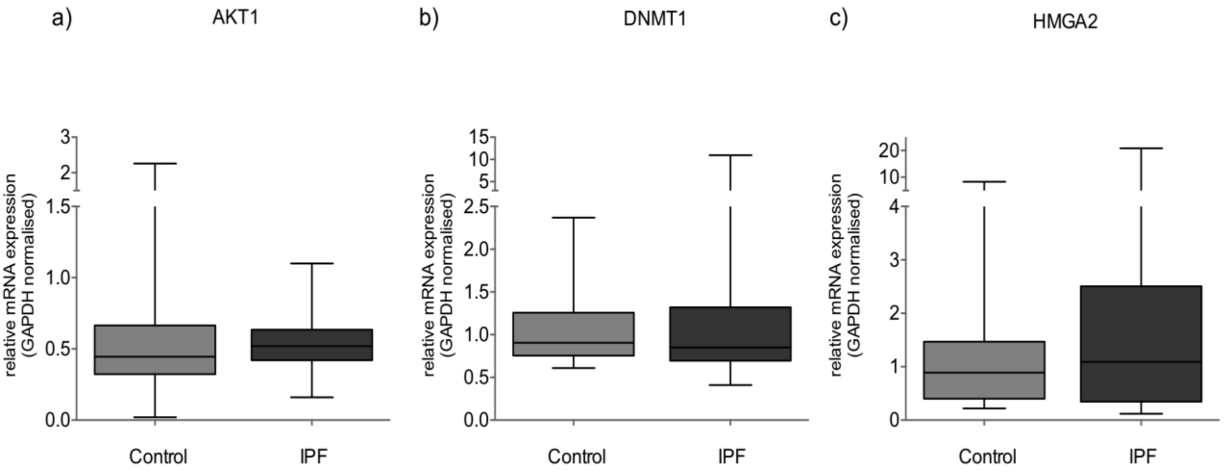

Figure S1.

Expression of a) AKT1 b) DNMT1, c) HMGA2 mRNA, normalized by GAPDH in control and IPF samples. Box plots represent median, 25 and 75 percentiles and error bars correspond to minimum and maximum values.

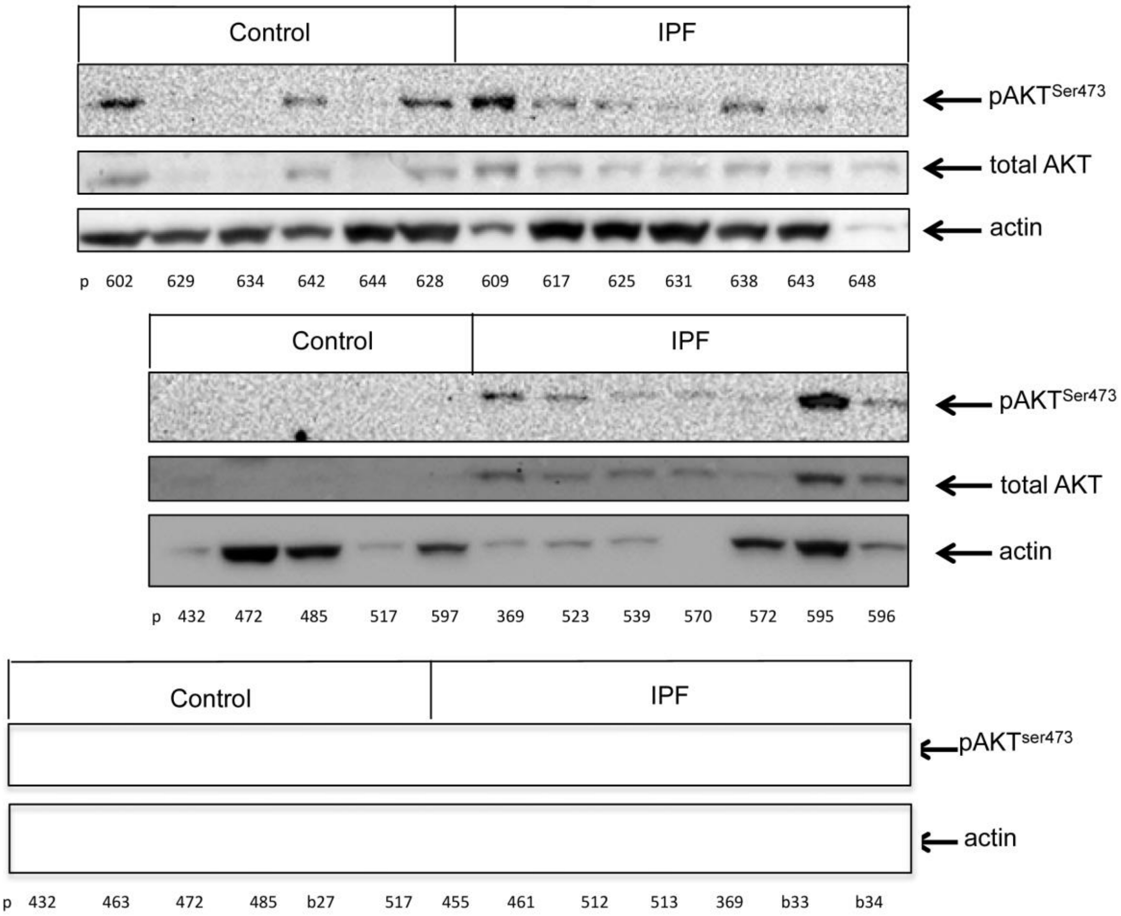

Figure S2. Western blot analyses of IPF and control BAL samples for pAKT^{Ser473} , total AKT and actin. Control and IPF groups are indicated above each blot and individual patient samples IDs are highlighted below each plot.

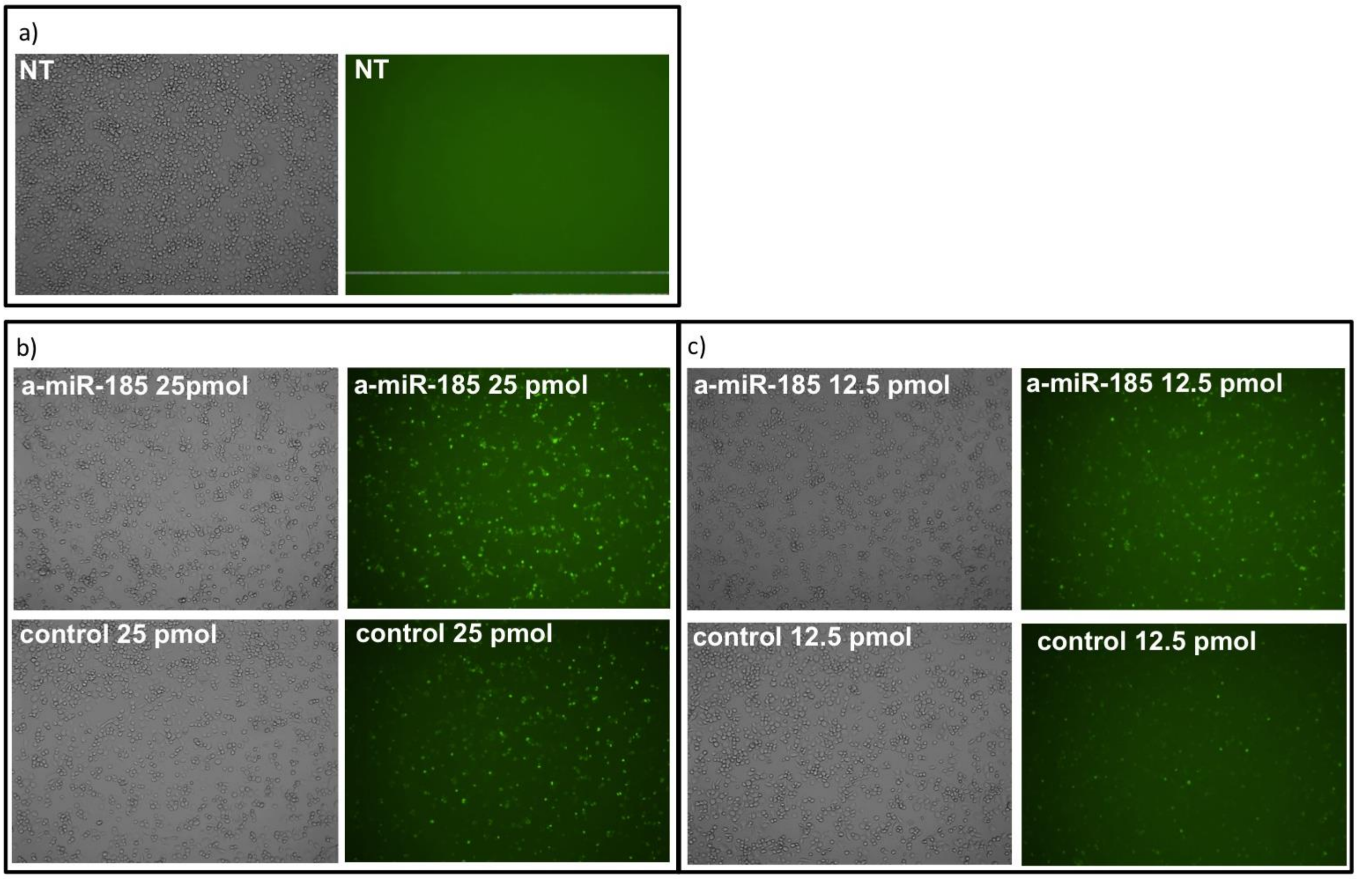

Figure S3. PMA treated THP1 cells examined under fluorescent microscope. a) Not transfected, b) transfected with 25 pico-molar and c) 12.5 pico-molar FITC-conjugated antagomir-185 or control RNAs.

Figure S3

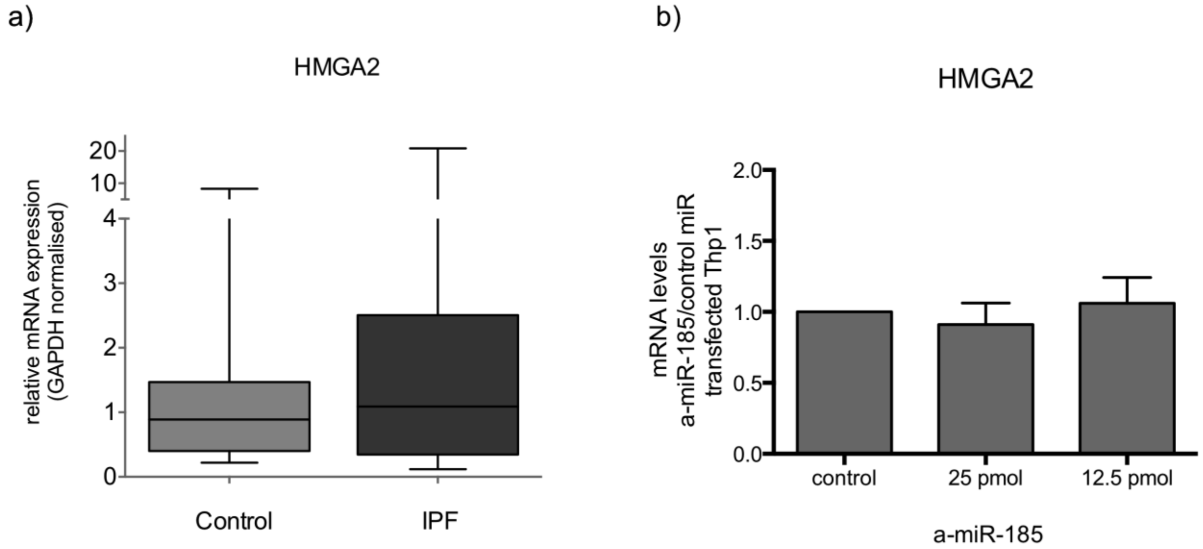

Figure s3. a) Expression of HMGA2 mRNA, normalized by GAPDH in control and IPF samples. b) fold change in expression of HMGA2 mRNA in 25 or 12.5 pico-molar concentrations of antagomiR-185 RNA transfected THP1 cells relative to control miR.
